# Surgical repair of left ventricular free-wall rupture complicating acute myocardial infarction: a single-center 30 years of experience

**DOI:** 10.3389/fcvm.2023.1348981

**Published:** 2024-01-10

**Authors:** Matteo Matteucci, Sandro Ferrarese, Vittorio Mantovani, Claudio Corazzari, Giangiuseppe Cappabianca, Corinne Messina, Sara Garis, Paolo Severgnini, Roberto Lorusso, Andrea Musazzi

**Affiliations:** ^1^ Department of Cardiothoracic Surgery, Heart and Vascular Centre, Maastricht University Medical Centre, Maastricht, Netherlands; ^2^ Cardiac Surgery Unit, ASST dei Sette Laghi, Department of Medicine and Surgery, University of Insubria, Varese, Italy; ^3^ Department of Biotechnology and Life Sciences, University of Insubria, Varese, Italy

**Keywords:** left ventricular free-wall rupture, acute myocardial infarction, surgical repair, mechanical complications, cardiac rupture

## Abstract

**Background:**

Left ventricular free-wall rupture (LVFWR) is a catastrophic complication of acute myocardial infarction (AMI). Historically, cardiac surgery is considered the treatment of choice. However, because of the rarity of this entity, little is known regarding the efficacy and safety of surgical treatment for post-infarction LVFWR. The aim of this study was to report a single-center experience in this field over a period of 30 years.

**Methods:**

Patients who developed LVFWR following AMI and underwent surgical repair at our Institution from January 1990 to December 2019 were considered. The primary end-point was in-hospital morality rate; secondary outcomes were long-term survival and postoperative complications. Multivariate analysis was carried out by constructing a logistic regression model to identify risk factors for early mortality.

**Results:**

A total of 35 patients were enrolled in this study. The mean age was 68.9 years; 65.7% were male. The oozing type of LVFWR was encountered in 29 individuals, and the blowout type in 6 subjects. Sutured repair was used in 77.1% of patients, and sutureless repair in the remaining cases. The in-hospital mortality rate was 28.6%. Low cardiac output syndrome was the main cause of postoperative death. Multivariable analysis identified age >75 years at operation, preoperative cardiac arrest, concurrent ventricular septal rupture (VSR) as independent predictors of in-hospital death. Follow-up was complete in 100% of patients who survived surgery (mean follow-up: 9.3 ± 7.8 years); among the survivors, 16 patients died during the follow-up with a 3-year and 12-year overall survival rate of 82.5% and 55.2%, respectively.

**Conclusions:**

Surgical treatment of LVFWR following AMI is possible with acceptable in-hospital mortality and excellent long-term results. Advanced age, concurrent VSR and cardiac arrest at presentation are independent risk factors of poor early outcome.

## Introduction

Left ventricular free-wall rupture (LVFWR) is a major lethal complication of acute myocardial infarction (AMI). Prior to the percutaneous intervention (PCI) era, the incidence of LVFWR was as high as 6% with ST-elevation myocardial infarction (STEMI) (1). However, thanks to the widespread use of reperfusion strategies for AMI, LVFWR has become increasingly uncommon, with recent literature reporting an incidence between 0.01% and 0.5% of myocardial infarction cases (2, 3). Most of the patients with LVFWR who survive the initial event, present with hemodynamic instability or cardiogenic shock. There is general consensus that surgical treatment is the only effective and durable therapeutic option. Several studies have reported the in-hospital results following surgical correction, however, since most of these analyses were performed over a relatively short time period, long-term outcome remain unclear. This report describes our experience in the surgical treatment of post-infarction LVFWR over a 30-year period.

## Methods

### Patients

In this retrospective study, we included 35 adult subjects who developed LVFWR complicating AMI and received surgical treatment at our hospital from January 1990 to December 2019. Patients’ clinical characteristics and operative information were collected from medical records through a standardized data collection form. Individuals with ventricular ruptures not AMI-related were excluded. Follow-up information was acquired from the Regional Institutional Health Database System. This study was performed in line with the principles of the Declaration of Helsinki and the protocol was approved by the institutional ethics committee. The requirement for informed consent was waived considering the retrospective study design.

### Definitions and end points

In accordance with our previous publication, blowout LVFWR was considered an abrupted rupture (with acute course) characterized by active bleeding and a macroscopic tear in the infarcted myocardium; while oozing LVFWR was considered an incomplete rupture (with subacute presentation) characterized by epicardial extravasation or slow bleeding which may be temporarily sealed by clot or pericardial adhesion (4). Sutureless technique (STL) was considered when LVFWR surgical treatment was accomplished using a collagen sponge or pericardium patch fixed on epicardium with surgical adhesive; while sutured technique (ST) was considered when the repair was performed using stitches or continuous sutures to close the myocardial tear or to secure a patch on the myocardium.

The primary end-point was in-hospital mortality. Secondary outcomes included long-term survival, postoperative complications and identification of prognostic factors associated with in-hospital mortality. We also assessed the variations of early mortality over the study period. To set the ideal threshold for the comparative periods, we identified the year of the first sutureless repair adoption in our center (i.e., 2003), to analyze whether such a change in the surgical technique had an impact on in-hospital mortality. Therefore, the two time frames compared for in-hospital outcomes were 1990–2002 and 2003–2019.

### Statistical analysis

Categorical variables were represented using frequencies and percentages (%), and the Chi-square test or the Fisher exact test was used to determine the difference between two groups. For continuous variables, the mean (standard deviation, SD) was used for description, and the Student *t*-test was used to compare the difference between groups. Variables that demonstrated a *p*-value < 0.2 in the univariate analysis were tested using multivariable analysis by forward stepwise logistic regression with the aim to identify independent predictors of in-hospital mortality. Survival curves were constructed with the Kaplan–Meier method and compared using the long-rank test. All the analyses were performed using the software package SPSS 25.0 for Windows (IBM, Chicago, USA). The level of significance was set at *p*-value < 0.05.

## Results

### Baseline characteristics

A total of 35 individuals underwent surgical repair for post-AMI LVFWR during the study period. The average age of patients was 68.9 ± 8.6 year, and female accounted for 34.3% of cases. The most common cardiovascular risk factors was high blood pressure, followed by diabetes mellitus and history of smoke. The mean time between AMI and cardiac rupture was 2.9 ± 2.4 days. Five subjects had been treated with thrombolysis for STEMI and 7 received percutaneous transluminal coronary angioplasty. In most of cases the diagnosis of LVFWR was made first by colour doppler echocardiography. Eighty percent of patients underwent coronary angiography; multivessel coronary artery disease was present in 18 individuals and single-vessel disease in 10 subjects. Most of the patients developed cardiogenic shock before surgery, whereas 7 subjects presented with cardiac arrest. Surgical status was listed as emergent or salvage in all individuals. Nearly 40% of subjects were supported preoperatively with an intra-aortic balloon pump (IABP), no patients received extracorporeal membrane oxygenation (ECMO) support. Preoperative clinical data are outlined in Table 1.

**Table 1:** Baseline and preoperative characteristics.

Variables	Patients (*n* = 35)	Survivors (*n* = 25)	Non-survivors (*n* = 10)	*p*-value
Age (year)	68.9 ± 8.6	67.3 ± 8.4	73 ± 8	*0*.*076*
Age >75 years	11 (31.4%)	5 (20%)	6 (60%)	*0*.*057*
Male (*n*)	23 (65.7%)	18 (72%)	5 (50%)	0.398
Hypertension (*n*)	15 (42.8%)	11 (44%)	4 (40%)	0.871
Diabetes (*n*)	13 (37.1%)	10 (40%)	3 (30%)	0.868
Smoker (*n*)	13 (37.1%)	10 (40%)	3 (30%)	0.868
PAD (*n*)	8 (22.8%)	4 (16%)	4 (40%)	0.279
Cardiogenic shock (*n*)	28 (80%)	20 (80%)	8 (80%)	0.640
Cardiac arrest (*n*)	7 (20%)	1 (4%)	6 (60%)	*0*.*001*
Pericardial tamponade (*n*)	21 (84%)	14 (56%)	7 (70%)	0.702
Pre-op IABP (*n*)	13 (37.1%)	8 (32%)	5 (50%)	0.543
Thrombolysis (*n*)	5 (14.3%)	3 (12%)	2 (20%)	0.939
PCI (*n*)	7 (20%)	6 (24%)	1 (10%)	0.640
Pericardiocentesis (*n*)	8 (22.8%)	7 (28%)	1 (10%)	0.484

Data are shown as mean ± standard deviation or *n* (%) as appropriate.

Italic values indicate significant *p*-values < 0.05.

n, number; IABP, intra-aortic balloon pump; PCI, percutaneous coronary intervention.

### Operative information

**Figure 1:**
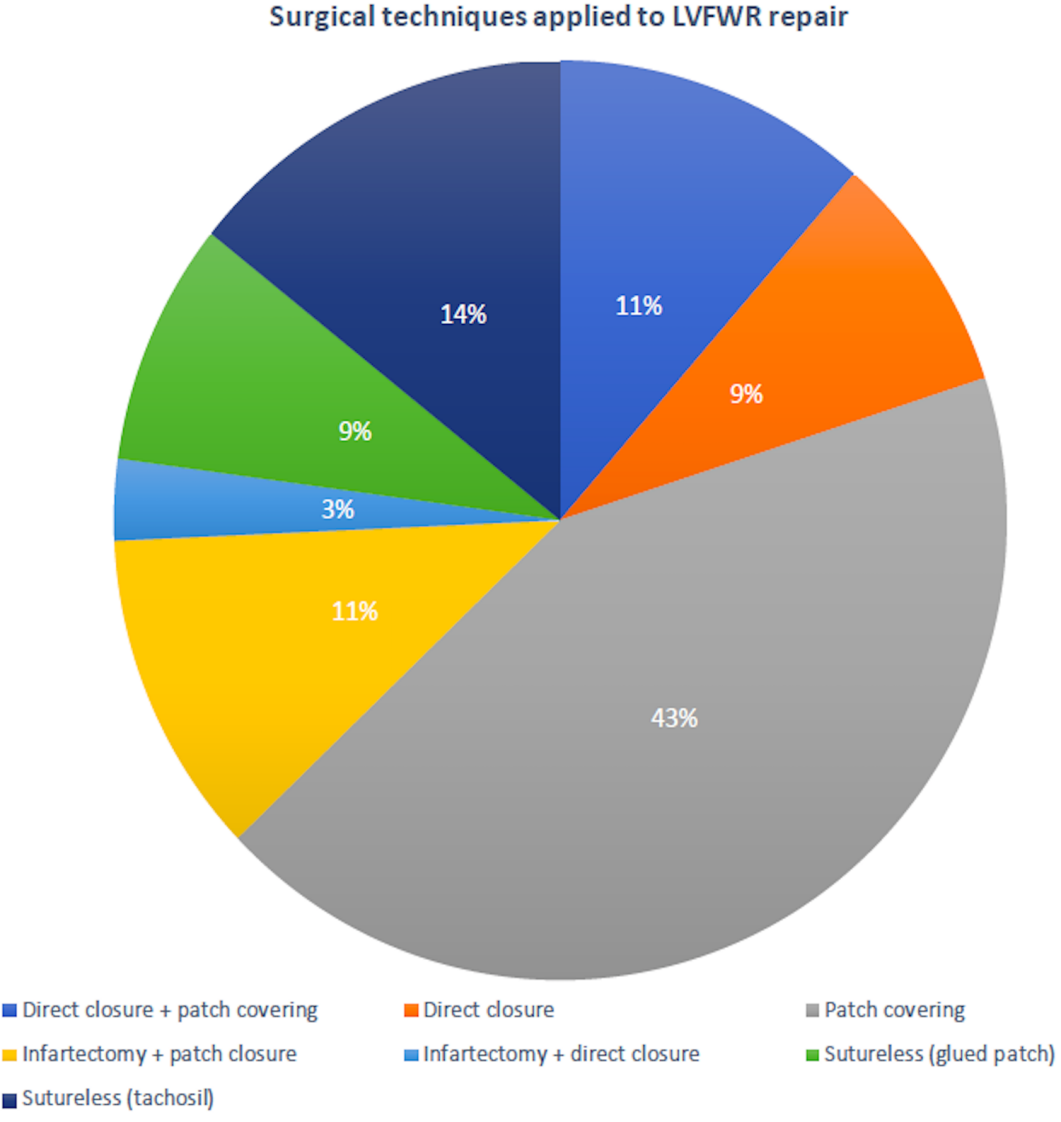
Surgical techniques used to repair the post-infarction left ventricular free-wall rupture.

Surgical procedures were performed through a standard median sternotomy. Almost all patients were operated on cardiopulmonary bypass (CPB); in only 2 cases, LVFWR repair was achieved on beating heart without CPB. Mean CPB time was 112.7 ± 61.5 min and aortic cross clamp duration was 69.5 ± 37.2 min. Oozing type was the rupture most commonly encountered. The LVFWR locations were in the antero-lateral wall in 12 patients (34.3%), in the lateral wall in 7 (20%) and in the postero-lateral wall in 16 (45.7%). A sutured technique was used in 77.1% of subjects to repair the ventricular rupture; in the remaining cases a sutureless technique was applied (Figure 1). Concomitant coronary artery bypass grafting (CABG) was accomplished in nearly 50% of patients and additive ventricular septal rupture (VSR) closure in 5 subjects. Postoperatively, almost one third of individuals required IABP support. Operative information and data are presented in Table 2.

**Table 2:** Perioperative characteristics.

Variables	Patients (*n* = 35)	Survivors (*n* = 25)	Non-survivors (*n* = 10)	*p*-value
Blowout rupture (*n*)	6 (17.1%)	4 (16%)	2 (20%)	0.831
Oozing rupture (*n*)	29 (82.8%)	21 (84%)	8 (80%)	0.831
Sutured technique (*n*)	27 (77.1%)	19 (76%)	8 (80%)	0.849
Sutureless technique (*n*)	8 (22.8%)	6 (24%)	2 (20%)	0.849
Concomitant CABG (*n*)	17 (48.5%)	12 (48%)	5 (50%)	0.789
CPB time (min)	112.7 ± 61.5	108.9 ± 51.9	121.5 ± 82.1	0.596
Cross-clamp time (min)	69.5 ± 37.2	67.3 ± 38.9	75.8 ± 33.5	0.609
Additive VSD repair (*n*)	5 (14.3%)	1 (4%)	4 (40%)	*0*.*027*
Post-op IABP (*n*)	12 (34.3%)	8 (32%)	4 (40%)	0.955

Data are shown as mean ± standard deviation or *n* (%) as appropriate.

Italic values indicate significant *p*-values < 0.05.

n, number; CPB, cardiopulmonary bypass; CABG, coronary artery bypass grafting; min, minutes; IABP, intra-aortic balloon pump; VSR, ventricular septal rupture.

### Early outcomes

In-hospital postoperative outcomes are shown in Table 3. The most common complications recognized following surgery were low cardiac output syndrome (LCOS) and atrial fibrillation, followed by bleeding requiring rethoracotomy and sepsis. Three patients were reoperated for LVFW re-rupture; one patients suffered papillary muscle rupture 7 days after LVFWR repair and underwent mitral valve replacement, whereas one subject was reoperated after 1 day for subsequent VSR. Overall, in hospital mortality was 28.6% (*n* = 10), with 2 intraoperative death due to irreparable cardiac rupture and biventricular failure precluding CPB weaning. LCOS with associated multiorgan failure (MOF) (*n* = 4) and kidney injury (*n* = 2) were by far the most frequent causes of in-hospital mortality; remaining causes included LVFW re-rupture (*n* = 1) and septicemia (*n* = 1). The mean duration of postoperative stay for hospital survivors was 17.6 ± 16.4 days; pre-discharge echocardiography revealed a mean left ventricular ejection faction (LVEF) of 38.3 ± 7.5%.

**Table 3:** Postoperative early outcomes.

Variable	Patients (*n* = 35)
Low cardiac output syndrome	10 (28.6%)
Atrial fibrillation	9 (25.7%)
Bleeding	5 (14.3%)
Sepsis	4 (11.4%)
AKI	3 (8.6%)
Reoperation for LVFW re-rupture	3 (8.6%)
Subsequent PMR or VSR	2 (5.7%)
Pneumonia	2 (5.7%)
On-table death	2 (5.7%)
In-hospital mortality	10 (28.6%)
Hospital stay (days)^a^	17.6 ± 16.4
Pre-discharge LVEF (%)^a^	38.3 ± 7.5

Data are shown as mean ± standard deviation or *n* (%) as appropriate.

AKI, acute kidney injury; LVFW, left ventricular free-wall; PMR, papillary muscle rupture; VSR, ventricular septa rupture; LVEF, left ventricular ejection fraction.

^a^ Hospital survivors.

**Figure 2:**
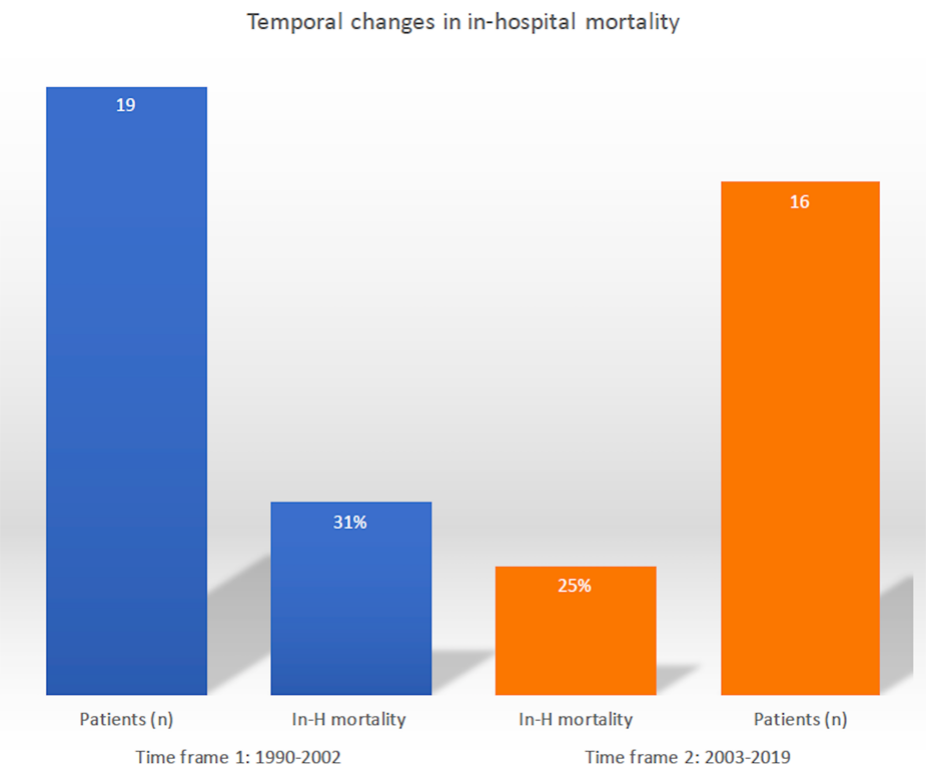
Temporal trend evaluation of in-hospital mortality following post-infarction left ventricular free-wall rupture surgical repair.

Temporal trend analysis demonstrated no substantial changes over time in the early mortality rate: 31% (time frame 1) vs. 25% (time frame 2) (Figure 2).

At univariate analysis, older age (*p *= 0.076), age >75 years (*p *= 0.057), cardiac arrest at presentation (*p *= 0.001) and concomitant VSR (*p *= 0.027) were associated with in-hospital mortality. However, multivariate analysis identified only age >75 years at operation (OR: 40.52, 95%CI: 1.206–1361.8; *p *= 0.049), preoperative cardiac arrest (OR: 91.45, 95%CI: 6.726–1243.5; *p *< 0.001) and concurrent VSR (OR: 25.71, 95%CI: 2.557–258.62; *p *= 0.006) as independent predictors of in-hospital death.

### Post-discharge survival

**Figure 3:**
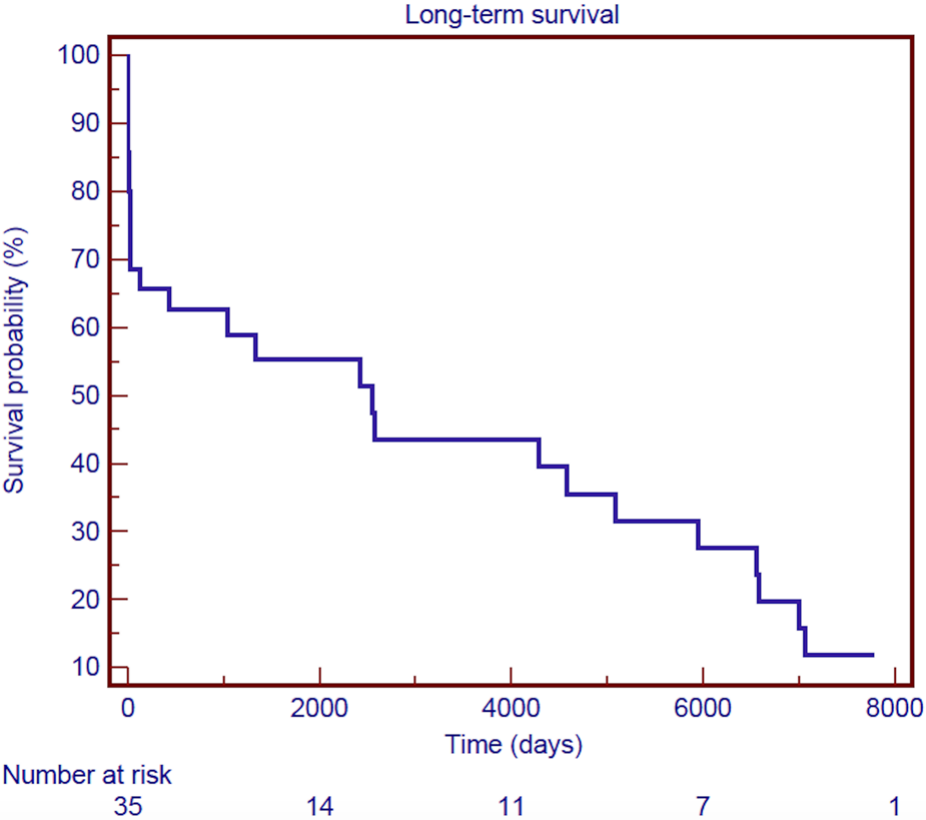
Kaplan–Meier survival curve of overall survival for all patients after surgical treatment of post-infarction left ventricular free-wall rupture.

**Figure 4:**
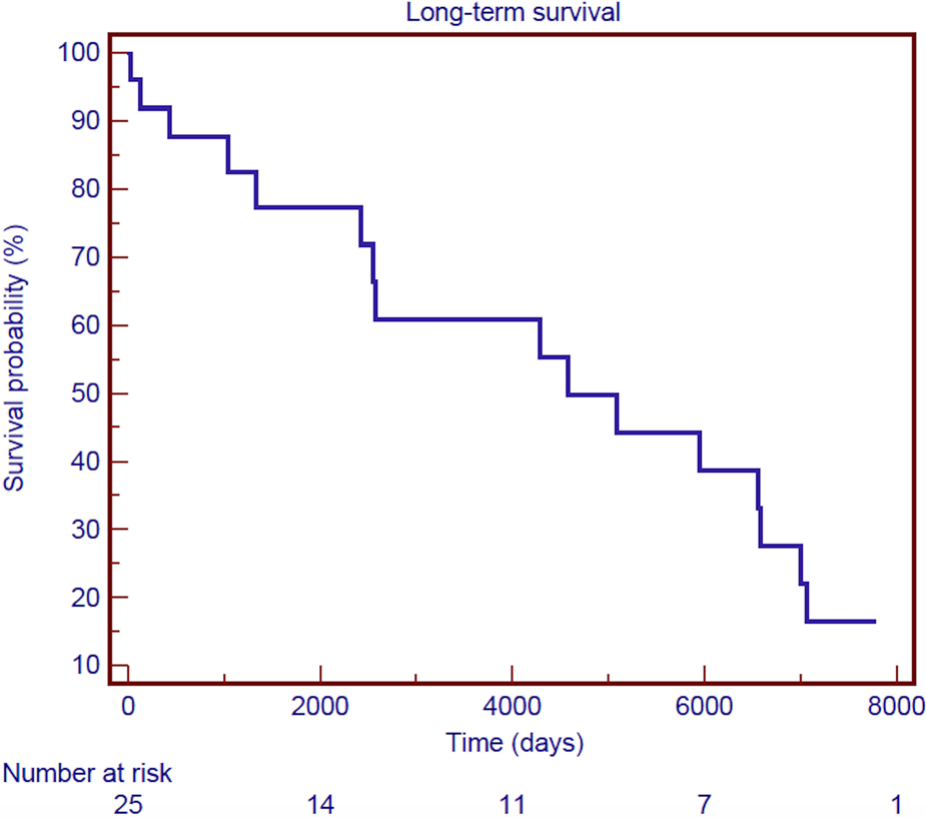
Kaplan–Meier survival curve of overall survival for hospital survivors after surgical treatment of post-infarction left ventricular free-wall rupture.

**Figure 5:**
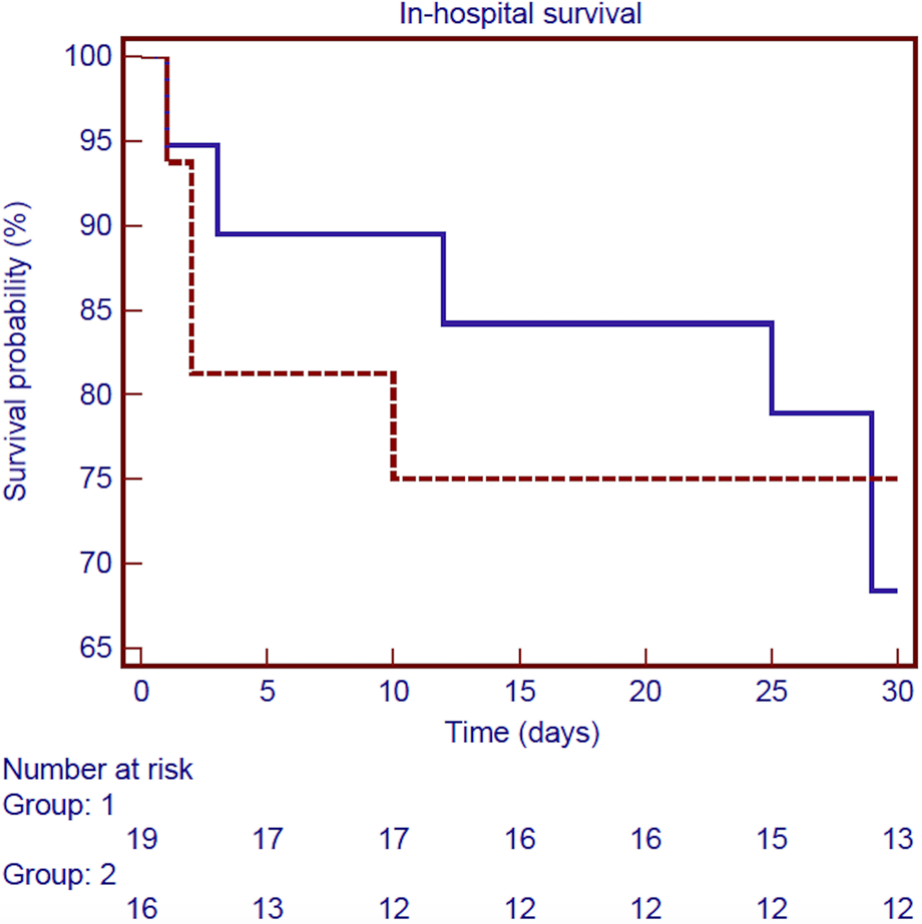
Kaplan–Meier survival curve of in-hospital survival for all patients according to the two different time frames considered: 1990–2002 (blue line) and 2003–2019 (red line) (long-rank, *p *= 0.711).

Follow-up was 100% for individuals who survived surgery. The mean follow-up time was 9.3 ± 7.8 years. Figure 3 shows the Kaplan–Meier estimate of overall survival (OS) including non-hospital survivors; OS rates at 3- and 12-years were 58.9% and 39.5%, respectively. Among the hospital survivors, 16 patients died during the follow-up with a 3- and 12-years overall survival rate of 82.5% and 55.2%, respectively (Figure 4). Figure 5 demonstrates no significant difference in the 5-year OS rates of all patients between the two different time frames compared (long-rank, *p *= 0.711).

## Discussion

Over the past two decades, the systematic adoption of early PCI for patients with AMI has had a favorable impact on the global incidence of LVFWR. The reduction in the rate of LVFWR achieved by PCI is probably related to the earlier and more effective restoration of coronary recanalization, compared with thrombolysis. Despite these improvements, mortality for patients who develop this post-AMI has remained high with no change over time (2). In this retrospective study we evaluated the outcomes of 35 patients surgically treated for post-infarction LVFWR at our institution between 1990 and 2019. The key findings were as follow: (i) relative high in-hospital mortality (∼30%) that remain substantially unchanged over the study period; (ii) excellent long-term survival for hospital survivors (3-year survival rate of ∼83%); age >75 years, cardiac arrest and concomitant VSR being the factors associated with an increased risk of early death.

Patients with LVFWR may present with chest pain, cardiogenic shock or cardiac arrest depending on the type of the rupture occurred (4). Large myocardial tear usually lead to sudden cardiac tamponade and electromechanical dissociation, whereas smaller and more gradual tear may be limited by clots formation with varying severity of symptoms (5). These two patterns are described in the literature as the blowout and oozing types, respectively (4). In accordance with previous reports, we noted a oozing-type rupture preponderance (6, 7). This observation may be explained by the fact that blowout ruptures are fatal in few minutes, whereas oozing cases are often subacute in nature presenting a window of opportunity for intervention. The literature presents diverging observations on the most common localization of LVFWR. While Iemura et al. reported that the anterior wall was more susceptible (8), Canovas and Colleagues reported that the rupture is more frequent on the lateral or posterior wall (9). In a recent publication, Formica et al. demonstrated that most of the ruptures were in the non-anterior localization (6); our findings of 34% anterior ruptures were consistent with this.

Non-surgical options for post-infarction LVFWR management include the installation of thrombin or fibrin glue injection into the pericardial space (4). However, surgical treatment remains the standard of care. Since the first successful repair reported by Fitzgibbon et al. (10), several different surgical techniques have been proposed over the time to treat the cardiac rupture. Initially, the ST were the only ones used; recently, the availability of collagen sponges or surgical biological glues have allowed the wide diffusion of the STL. To date, which technique is the most appropriate is still controversial. In a recent systematic review, the two surgical methods showed comparable early mortality (11). Our results seem to be in accordance with this observation; indeed, we did not find a significant difference in terms of in-hospital mortality between the two techniques (29% for ST vs. 25% for STL). However, in our cohort only a small number of patients were treated with a STL technique, therefore more consistence data are needed to assess whether one method is superior (or not) over the other.

The advantage of performing concomitant CABG during emergency LVFWR repair remains debated. We generally do not revascularize the culprit vessel in the infarcted region associated with the myocardial rupture, whereas other coronary lesions are often grafted at the time of surgery. We, like other authors (6), did not find a beneficial effect of simultaneous CABG on early mortality. We can postulate that the real effectiveness of the myocardial surgical revascularization is underestimated by the low number of subjects who had undergone additive CABG (<50%). Further analysis are required to evaluated the effectiveness of concomitant CABG in this context.

The mortality rates for medically treated individuals are awfully high at up to 90% (12). By contrast, those undergoing surgery have more favorable outcomes. Our in-hospital mortality was 28.6%, which is consistent with the 12%–36% mortality seen in the studies present in literature (6–9, 13, 14). Risk factors associated with increased mortality have been assessed in several studies and include female sex, cardiac arrest at presentation, impaired left ventricular function and the need of preoperative extracorporeal membrane oxygenation (6, 7, 13). The risk factor that has been reported to have the strongest association with early mortality is preoperative critical status (6, 7, 13), reflecting the high-acuity of this post-AMI complication. In agreement with other studies (6, 7, 13), cardiac arrest at presentation was found to significantly increase the risk of in-hospital death in this analysis. Such an observations highlight the need for early recognition and urgent treatment to mitigate prolonged state of end-organ hypoperfusion and potential death.

Despite advances in diagnostic and surgical procedures for LVFWR, we found that in-hospital mortality rate remained high and substantially unchanged over time. The reason for the luck of decrease in mortality is not clear. The rarity of the condition and the associated lack of expertise represent the major challenge to the surgeon. Moreover, the abrupt and often unpredictable hemodynamic deterioration imposed by this post-AMI event may overwhelm any available therapeutic resources. The use of MCS for LVFWR represents a new trend in management (15). Preoperatively, in patients presenting with cardiac arrest or cardiogenic shock, MCS implantation allows immediate circulatory support providing time and circulatory stabilization for the diagnostic workup and surgical repair. In the immediate postoperative period, MCS would allow for ventricular recovery and limit the development of LCOS. In our experience, 40% of patients died perioperatively due to LCOS. It is noteworthy that no patients were supported postoperatively (neither preoperatively) with ECMO. These findings seem to indicate the need for an extended and more aggressive adoption of mechanical circulatory support in those individuals. However, it remains to be verified whether wider use of MCS devices will decrease mortality in this setting. Further and dedicated studies are warranted to provide additional and more consistent data.

In literature, only few reports describe the long-term survival of patients operated for LVFWR. In the current study, the 3-year and 12-year OS rate was 58.9% and 39.5%. These survival rates are comparable to those reported by others (6, 13). Besides, it’s interesting to underline that when survival analysis is limited to patients discharged from the hospital only, life-expectancy is excellent approaching 83% at 3 years and 56% at 12 years.

### Limitations

Several limitations of the current study should be cmentioned. This was a single-center experience; therefore, the number of subjects should be considered too low to gain definitive conclusions. Because of the retrospective nature of the study, both the presence of selection bias and confounders can not be excluded. Because of the unavailability of data on cause of late death, only all-cause mortality have been assessed for the long-term outcome. Our research may be considered as an attempt to shed some light on this rare post-AMI complication and encourage further analysis, rather than state definitive assumptions.

## Conclusions

LVFWR is a high-acuity and time-sensitive complication of AMI. The surgical repair is possible with acceptable in-hospital mortality and excellent life-expectancy, particularly for hospital survivors. Age >75 years, cardiac arrest at presentation and concomitant VSR are poor independent prognostic factors. Despite advances in medical practices and surgical techniques during the last decades, the early mortality rate for this serious post-infarction complication has not changed and continues to be a challenge for clinicians. Further analyses with larger number of patients are needed to corroborate these observations, and especially to assess whether more extensive use of temporary MCS may improve outcomes.

## Data Availability

The raw data supporting the conclusions of this article will be made available by the authors, without undue reservation.
